# Perinatal Depressive Symptoms and Viral Non-suppression Among a Prospective Cohort of Pregnant Women Living with HIV in Nigeria, Kenya, Uganda, and Tanzania

**DOI:** 10.1007/s10461-022-03810-6

**Published:** 2022-10-09

**Authors:** Tessa Concepcion, Jennifer Velloza, Christopher G. Kemp, Amritha Bhat, Ian M. Bennett, Deepa Rao, Christina S. Polyak, Julie A. Ake, Allahna Esber, Nicole Dear, Jonah Maswai, John Owuoth, Valentine Sing’oei, Emmanuel Bahemana, Michael Iroezindu, Hannah Kibuuka, Pamela Y. Collins

**Affiliations:** 1 Department of Global Health, University of Washington, Seattle, WA USA; 2 Department of Epidemiology & Biostatistics, University of California San Francisco, San Francisco, CA USA; 3 Department of International Health, Johns Hopkins University, Baltimore, MD USA; 4 Department of Psychiatry and Behavioral Sciences, University of Washington, Seattle, WA USA; 5 Department of Family Medicine, University of Washington, Seattle, WA USA; 6 U.S. Military HIV Research Program, Walter Reed Army Institute of Research, Silver Spring, MD USA; 7 Henry M. Jackson Foundation for the Advancement of Military Medicine, Inc., Bethesda, MD USA; 8 US Army Medical Research Directorate-Africa, Kericho, Kenya; 9 U.S. Army Medical Research Directorate-Africa, Kisumu, Kenya; 10 HJF Medical Research International, Kisumu, Kenya; 11 HJF Medical Research International, Mbeya, Tanzania; 12 HJF Medical Research International, Abuja, Nigeria; 13 Makerere University Walter Reed Project, Kampala, Uganda

**Keywords:** HIV/AIDS, Depression, Perinatal, Pregnancy, Africa, Viral non-suppression

## Abstract

Depression is common during pregnancy and is associated with reduced adherence to HIV-related care, though little is known about perinatal trajectories of depression and viral suppression among women living with HIV (WLHV) in sub-Saharan Africa. We sought to assess any association between perinatal depressive symptoms and viral non-suppression among WLWH. Depressive symptomatology and viral load data were collected every 6 months from WLWH enrolled in the African Cohort Study (AFRICOS; January 2013–February 2020). Generalized estimating equations modeled associations between depressive symptoms [Center for Epidemiological Studies Depression (CES-D) ≥ 16] and viral non-suppression. Of 1722 WLWH, 248 (14.4%) had at least one pregnancy (291 total) and for 61 pregnancies (21.0%), women reported depressive symptoms (13.4% pre-conception, 7.6% pregnancy, 5.5% one-year postpartum). Depressive symptomatology was associated with increased odds of viral non-suppression (aOR 2.2; 95% CI 1.2–4.0, p = 0.011). Identification and treatment of depression among women with HIV may improve HIV outcomes for mothers.

## Introduction

Girls and women of childbearing age account for 59% of new HIV infections in sub-Saharan Africa (SSA) [1], and many women living with HIV (WLWH) intend to have children [2]. Fortunately, over the last two decades, the number of children born with HIV due to vertical transmission has decreased dramatically with public health measures such as increased uptake of safe and effective antiretroviral therapy (ART) for pregnant women [3–6]. Though effective HIV prevention and treatment interventions exist and are available in SSA [7, 8], dramatic gaps in uptake are reported, including loss to follow up (as high as 88% in some contexts) and poor adherence to ART [9], particularly among young women of reproductive age in SSA [10, 11]. HIV incidence among pregnant women at antenatal clinics ranges from 1.5% (South Africa) [12] to 3.3% (Western Kenya) [13]. The rate of vertical HIV transmission with no intervention (defined as the rate of children born with HIV that was acquired from their mother during pregnancy, childbirth, or breastfeeding) ranges between 13 and 48% [14, 15].

Depression is a common mental disorder that affects many WLWH and is associated with both ART adherence and pregnancy outcomes [14, 16–19]. WLWH have a significantly higher odds of depressive symptoms during the antenatal (odds ratio [OR] 1.42; 95% confidence interval [CI] 1.12–1.80) and postnatal (OR 1.58; 95% CI 1.08–2.32) periods (together termed the “perinatal period”), compared to pregnant women living without HIV [20]. Globally, studies estimate that between 25 to 50% of pregnant WLWH have antenatal depressive symptoms at time points in every trimester of pregnancy [15, 16, 20–23]. Depression and low social support can reduce ART adherence for pregnant WLWH [24], which can lead to viral non-suppression and increased risk of vertical transmission of HIV [25–27]. In addition to the behavioral link between depressive symptoms and viral load, depression has also been shown to be directly associated with viral load, even in cases of high adherence to ART, through an altered immune response [28–30]. Evidence suggests that treatment with antidepressant medication may reduce susceptibility to cellular infection [30, 31]. Additionally, pregnant WLWH facing mental health difficulties experience risk of medical sequelae for their newborns such as preterm birth, stillbirths, and babies born with low birthweight [32]. ART usage has shown mixed results in alleviating this risk [3, 32].

Studies of HIV and mental health among pregnant WLWH have largely focused on ART use and adherence as proxies for viral non-suppression [33–35]. While associations between self-report adherence to ART and viral load are generally strong among pregnant women, pharmacologic tests of viral non-suppression are more robust and allow for a more accurate assessment of risk for vertical transmission than adherence metrics alone [36, 37]. Viral load is an important clinical indicator of HIV care and the final target of the UNAIDS 90–90–90 [38]. Understanding whether depression directly affects viral non-suppression for pregnant WLWH would enable providers to target HIV and maternal health services to address depressive symptoms, reduce vertical HIV transmission, and improve child survival. In addition, gaps remain in understanding the impact of depression on viral load throughout the perinatal period, from pre-conception, through pregnancy, and postpartum. The primary objective of this analysis is to understand the association between depressive symptoms and viral non-suppression among pregnant WLWH in Nigeria, Kenya, Uganda, and Tanzania during their perinatal period. The secondary objective is to explore associations between depressive symptoms and viral non-suppression on birth outcomes, including preterm birth and pregnancy loss.

## Methods

### Study Design and Participants

The African Cohort Study (AFRICOS) is an ongoing prospective longitudinal cohort study enrolling people living with HIV (PLWH) at 12 HIV care clinical sites in Kenya, Tanzania, Uganda and Nigeria [34, 39–41]. These sites are maintained by five President's Emergency Plan for AIDS Relief (PEPFAR) programs. The primary objective of AFRICOS is to assess the impact of clinical practices, biological and socio-behavioral factors on HIV infection and disease progression in an African context. AFRICOS began enrollment in January 2013. The primary objective of the parent study, AFRICOS, is descriptive, and the hypotheses tested will be exploratory. Therefore, the enrollment targets for AFRICOS were determined by the relative size of each PEPFAR Program, research capacity at each site, and available resources.

Since 2013, AFRICOS has enrolled individuals aged 18 years or older with and without HIV, in an approximate 5:1 ratio. Participants living with HIV were invited from clinical site patient lists to participate in the study by random selection stratified by gender and ART status. PLWH were selected from current HIV clinic client lists, with the number of participants reflective of the proportion of PLWH observed in the clinic population. A small proportion (less than 9%) [42] were recruited from other HIV studies conducted locally. PLWH were eligible for AFRICOS if they were at least 18 years old, consented to data and specimen collection, and had ongoing receipt of HIV care at the enrolling clinical site. Notably, AFRICOS began to enroll 15- to 17-year-old participants in January 2020, but since this analysis only includes data up to February 2020, these participants are not included in this analysis. PLWH are selected to proportionally reflect the ratio of females to males and the ratio of participants on ART to those not on ART (current ART status at screening/enrollment) at each of the PEPFAR sites. This is accomplished through the use of clinic registries, and randomization is conducted in SAS (SAS Institute, Cary, North Carolina). Minimum viral load results at enrollment/baseline were not considered for randomization Participants are seen once every six months during their participation. Individuals who were pregnant at enrollment were excluded from AFRICOS but women who became pregnant during participation continued enrollment and were included in the cohort. Participants in previous PEPFAR vaccine, therapeutic and cohort studies at locations where AFRICOS enrollment occurs were eligible for enrollment into AFRICOS. Furthermore, enrollment in AFRICOS does not preclude enrollment into future vaccine, therapeutic or cohort studies. For this analysis, we examined the prospective relationship between pregnancy, depressive symptoms, HIV viral non-suppression, and child outcomes among WLWH. Participants were eligible for the present analysis if they were enrolled between January 2013 and February 2020 and reported a birth during the study period.

### Data Collection

Participants completed a medical history and physical examination, demographic and behavior questionnaire, and underwent phlebotomy at every visit. Enrolled participants were seen at study clinical sites once every six months. They were offered ART and prenatal care per each country’s national guidelines. Data are captured on paper case report forms and then entered and verified in the ClinPlus platform (Anju Software, Tempe, AZ). Data are stored on a secure server located at the U.S. Military HIV Research Program headquarters.

#### Demographics

Participant age, gender, and obstetric history were determined through chart review. Participant household income (i.e., “What is your household’s total income per week?”) and food insecurity (i.e., “Have you had enough food to eat over the past 12 months?”) were determined through subject questionnaires.

#### Household Income

Household income was reported at baseline via self-report. We converted the household incomes into Purchasing Power Parity (PPP) values for comparability. First, we converted local currency into USD for the year that the income was reported [43]. Then we multiplied by the price level ratio of PPP conversion factor (GDP) to market exchange rate for each country as reported by the World Bank for the respective year reported [44].

#### Perinatal Period

For our analysis we defined three phases: (1) pre-conception (52 weeks prior to estimated date of conception), (2) pregnancy (length of pregnancy in weeks), and (3) postpartum (52 weeks following birth). We selected these periods based on recognition that perinatal mood disorders are now defined as occurring in pregnancy and the 12 months postpartum [45–47]. Pregnancy was defined as the duration of gestational age of the baby in weeks. If gestational age was missing it was estimated to be 40 weeks since most births occur between 38 and 42 weeks [48]. Timepoints outlining these three time periods were calculated using the date of birth (DOB) of the baby and gestational age. DOB was recorded by chart review. If DOB was missing but age of child was reported at a subsequent visit, we backdated the age of the child from the visit date to estimate DOB. If no subsequent visit or DOB was recorded, the DOB was estimated to be the visit date that the mother first reported the birth outcome.

#### Depressive Symptoms

Depressive symptoms were evaluated using the Center for Epidemiologic Studies Depression (CES-D) scale [49], which was translated into Luganda, Luo, Pidgin English, and Kiswahili and performed as part of an interviewer-administered questionnaire. This scale has been previously validated among pregnant WLWH and HIV-uninfected pregnant women in Uganda [50]. The scale has also been validated among other populations in Uganda [51], Kenya [52], and Tanzania [52]. For this analysis, elevated depressive symptoms were defined as a CES-D score ≥ 16 and were our primary exposure. We also considered highly elevated depressive symptoms (CES-D ≥ 20) in secondary analyses. A cut-off score of 20 has been suggested as an optimal threshold for detection of major depression, minor depression, and dysthymia [53]. In our analysis, elevated or highly elevated depressive symptoms during a phase of the perinatal period were defined as at least one CES-D score above 16 or 20, respectively, during that phase. For example, if a participant had a score of 17 (elevated depressive symptoms) at one visit during the pre-conception phase, and a score of 8 (not elevated depressive symptoms) at the next visit during the same phase, the participant would be counted as having elevated depressive symptoms during the pre-conception phase. We also conducted secondary analyses where we defined elevated depressive symptoms as CES-D ≥ 10 [54].

#### Viral Load

Viral loads (plasma HIV RVA levels) were measured using the on-site, Roche COBAS® Taqman® HIV-1 or Abbott RealTime HIV-1 Viral Load assays. AFRICOS sites running viral load tests were all enrolled in external quality assessment (EQA); the EQA providers vary by site, typically with 2 to 3 testing cycles per year. All panel members are run and analyzed in the same manner as patient samples. Results are submitted to the EQA Provider and graded by the provider inclusive of peer review, and final report returned to site. The site reviews the final report for acceptability, monitors, tracks, and in cases of unacceptable results, completes an investigation along with corrective and preventative actions as needed. For the primary analysis, viral non-suppression was defined as a viral load of ≥ 200 copies per milliliter of blood. Viral non-suppression for a phase of the perinatal period was defined as at least one viral non-suppression result during that phase. We used 200 copies/ml as our cutoff to be in alignment with the Center for Disease Control and Prevention (CDC) definition of viral non-suppression [55] and to remain consistent with literature on conservative estimates of viral non-suppression among pregnant women [56, 57].

#### Birth Outcome and Gestational Age

Birth outcome data was collected through medical record chart review at each clinical site. Since data was recorded every 6 months, the same birth outcome could be reported at consecutive visits. In cases where the same birth was reported at multiple visits, data was merged to capture as many variables about a single birth as possible.

Gestational age data was collected via chart review and reported in units of days, weeks, or months. For analysis, all gestational ages were converted to gestational weeks. Where gestational age was not reported or missing, gestational weeks was assumed to be full term (40 weeks) for live births and still births and 20 weeks for spontaneous abortions or miscarriages.

Preterm birth is defined by WHO as babies born alive before 37 weeks of pregnancy are completed (through 37 weeks 6 days) [58]. For this analysis, “full-term” was defined as live births with a gestational age of greater than 37 completed weeks. “Moderate-to-late preterm” is defined as born between 32 and 36 completed weeks of pregnancy. “Very preterm” is defined as born at less than 32 weeks of pregnancy [58]. Other birth outcomes reported were stillbirths or spontaneous abortions and are referred to as pregnancy loss in this analysis. Birth outcomes were categorized as binary variables of (a) alive and full term or (b) pre-term or pregnancy loss.

### Statistical Analysis

We used descriptive statistics (frequencies, medians) to summarize demographic information among participants and the frequency of elevated depressive symptoms, viral non-suppression, and birth outcomes during the study period. Our baseline descriptive statistics used the total number of participants as the denominator. Other descriptive statistics use the total number of pregnancies as the denominator.

For our primary analysis, we were interested in exploring the effect of depressive symptoms (CES-D ≥ 16) on viral non-suppression (viral load ≥ 200 copies/ml) in WLWH who ever had a pregnancy during participation in AFRICOS. To answer this question, we constructed a visit-level dataset where each visit during a women’s perinatal period represented a unique record. We used generalized estimating equations (GEE) to model the association between depressive symptoms and viral non-suppression during the perinatal period. We fit the model using a logistic link, binomial distribution, and robust standard errors to estimate odds ratios while accounting for repeated measures over time and correlated demographic and behavioral data among a subset of mothers who were included more than once because they reported multiple births in the cohort. We included pregnancy phase (preconception, pregnancy, or postpartum), study site, and gravida a priori in our regression models*.* We also explored confounding by several additional covariates hypothesized to have a relationship with depressive symptoms and viral non-suppression, including literacy, food insecurity, PPP, highest education level, ARV medication type (EFV and/or NVP), and country. EFV and NVP medication types have been associated with depressive symptoms and suicidal ideation in previous studies [59, 60]. Our multivariable model included those covariates that we identified to be statistically significantly associated with viral non-suppression (any baseline factors associated with viral non-suppression with p-value < 0.10 were included in the multivariable models). Retrospective power calculations estimated the power based on the sample size, 0.05 Type I error rate, and odds ratios of viral non-suppression by depressive symptom status [61].

In addition to this primary analysis, we conducted several sensitivity analyses by altering our exposure and outcome definitions and sample selection criteria for our GEE models. Specifically, these sensitivity analyses included: (1) using self-reported ART adherence as our outcome variable in place of viral non-suppression, with any self-reported missed doses in the past one month categorized as non-adherence; (2) using a CES-D score of ≥ 10 as our exposure variable to capture a more sensitive depressive symptom measurement; (3) using a CES-D score of ≥ 20 as our exposure variable to capture a more specific depressive symptom measurement; and (4) restricting the analysis to mothers who had live births. In the second and third sensitivity analyses, we included a lower and higher threshold for elevated depressive symptoms to evaluate a dose–response relationship between our primary predictor (depressive symptoms) and outcome (viral non-suppression). We expected to see a smaller and higher magnitude of associations for the lower and higher thresholds, respectively.

We were also interested in exploring the associations between depressive symptoms, viral non-suppression, and birth outcomes. For this analysis, a birth-level dataset was used to run logistic regressions for depressive symptoms, viral non-suppression, and birth outcome. Analyses were conducted using SPSS (IBM Corp. Released 2020. IBM SPSS Statistics for Windows, Version 27.0. Armonk, NY: IBM Corp).

### Ethical Consideration

All participants provided written informed consent. The institutional review boards of the Walter Reed Army Institute of Research, Makerere University School of Public Health, Kenya Medical Research Institute, Tanzania National Institute of Medical Research, and Nigerian Ministry of Defense approved the study. This analysis was conducted using an anonymized dataset.

## Results

From January 2013 to February 2020, 1722 WLWH were enrolled in the AFRICOS study. Of WLWH, 248 (14.4%) had at least one pregnancy after enrollment; these 248 women reported 291 total pregnancies. One woman (0.4%) recorded four pregnancies, four women (1.6%) recorded three pregnancies, 32 women (12.9%) recorded two pregnancies, and 211 women (85.1%) recorded one pregnancy. The majority of women lived in Kenya (44.8%), followed by Uganda (25.4%), Nigeria (17.3%), and Tanzania (12.5%). Most women were married or living with their partner (61.7%) and the median number of previous pregnancies was 4. About 50% of the women had completed primary school and just over one third had completed secondary school (34.3%). There were 50 pregnancy losses comprising 42 spontaneous abortions and 8 stillbirths. One child (0.3%) was confirmed to have acquired HIV through vertical transmission, 68 children (23.4%) were confirmed HIV-uninfected, and 222 children (76.3%) had an unknown or not reported HIV status at the time of chart review. Approximately 21.0% of women had depressive symptoms at their enrollment visit (Table 1).

**Table 1:** Demographics of WLWH (n = 248) who had a pregnancy during participation (n = 291)

Baseline^a^ characteristics of mothers (n = 248)	Frequency^b^	
Country		
Kenya	111	(44.8)
Nigeria	43	(17.3)
Tanzania	31	(12.5)
Uganda	63	(25.4)
Marital status		
Single	49	(19.8)
Married or living with partner	153	(61.7)
Divorced/separated	32	(12.9)
Widowed	14	(5.6)
Income (PPP) (median, IQR)	3.8	(1.2–11.4)
Gravida (median, IQR)	4.0	(3.0–5.0)
Food insecurity	76	(30.6)
Literacy (none)	29	(11.7)
Education level		
No schooling	9	(3.6)
Primary school	124	(50.0)
Secondary school	85	(34.3)
Post-secondary school	30	(12.1)
CES-D ≥ 16 at baseline	52	(21.0)
Number of years diagnosed with HIV prior to first birth in study (median, IQR)	3.9	(1.6–6.9)

Birth-related characteristics (n = 291)		
Mother’s age at delivery (median, IQR)	31.0	(27.0–36.0)
Gestational weeks at delivery (median, IQR)	39.1	(39.1–39.1)
Birth outcome		
Alive & full term	219	(75.3)
Pre-term or not-surviving	72	(24.7)
Moderate to late preterm^c^	15	(5.2)
Very preterm^c^	7	(2.4)
Extremely preterm^c^	0	(0.0)
Pregnancy loss^c^	50	(17.2)

^a^Baseline data reflects visit 1 enrollment for each participant, not their first visit of the perinatal period

^b^Frequency is reported as N (%) for categorical variables and median (IQR) for continuous variables

^c^Percent of all pregnancies

A total of 106 women (36.4% of all 291 pregnancies) completed all study visits during their perinatal period (5 or 6 visits depending on pregnancy duration and visit timing), 108 women (37.1%) missed 1 study visit, 58 women (19.9%) missed 2 study visits, 17 women (5.8%) missed 3 study visits, and 2 women (0.7%) missed 4 study visits. Among women who did not miss their visit, only one did not have a CESD reported at their visit (postpartum) and 19 were missing a viral load result at their visit (4 in pre-conception, 1 in pregnancy, and 14 in postpartum). Due to missed visits and incomplete data at study visits, a total of 81.1% of women had a CES-D score and 79.4% of women had a viral load value for all perinatal periods. DOB was missing for 127 children (43.6%) and of these children, 63 (49.6%) had a reported age at the study visit which allowed us to estimate their DOB. Sixty-four children (22.0%) were missing both DOB and estimated age. Only 17 children (5.8%) were missing their gestational age at birth. Gestational age was missing for two live births, four still births, and 11 spontaneous abortions or miscarriages.

### Associations Between Depressive Symptoms and Viral Non-suppression

A total of 1263 participant visits were included in the analysis of associations between depressive symptoms and viral non-suppression during the perinatal period. Elevated depressive symptoms (CES-D ≥ 16) were reported at least once among 61 (21.0%) of the 291 pregnancies. Of those who reported depressive symptoms, 45 (74%) had elevated symptoms at one visit, 12 (20%) had elevated symptoms at two visits, 3 (5%) had elevated symptoms at three visits, and 1 (2%) had elevated symptoms at four visits during the period.

**Fig. 1:**
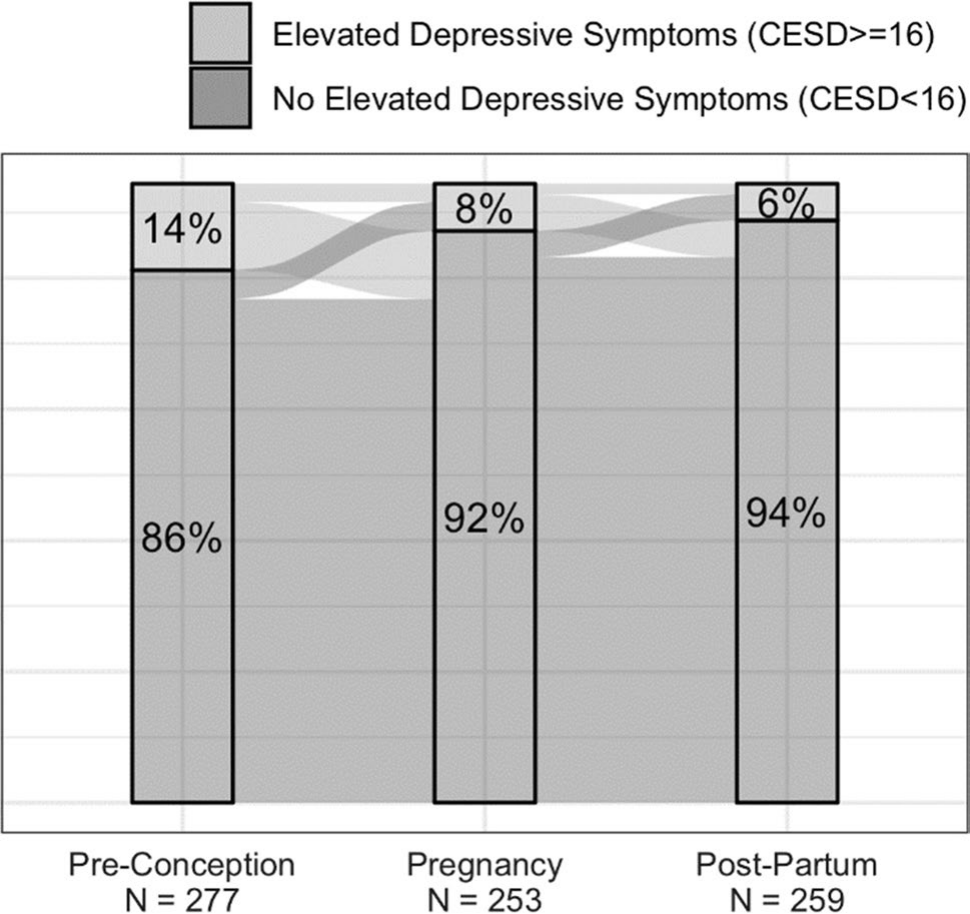
Trends in depressive symptoms during perinatal period

Throughout the 1-year pre-conception phase, 39 women (13.4%) had elevated, and 25 women (8.6%) had highly elevated depressive symptoms. During pregnancy, 22 women (7.6%) had elevated, and 9 women (3.1%) had highly elevated depressive symptoms. For 1 year postpartum, 16 women (5.5%) had elevated, and 13 women (4.5%) had highly elevated depressive symptoms (Fig. 1). A total of 93 women (32.0%) were virally non-suppressed at least once during one-year pre-conception, 57 women (19.6%) during pregnancy, and 26 women (8.9%) during one-year postpartum.

In our primary, visit-level, multivariable analysis, elevated depressive symptoms (CES-D ≥ 16) were associated with an increased odds of viral non-suppression compared to participants with lower symptom scores, after adjusting for perinatal period (aOR 2.2; 95% confidence interval [CI] 1.2–4.0, p = 0.011) (Table 2). The pre-conception and pregnancy phases of the perinatal period were also associated with an increased odds of viral non-suppression when compared to the post-partum phase (aOR 4.7 and 2.7, respectively; 95% CI 3.1–7.3 and 1.8–4.3, p ≤ 0.001 and < 0.001, respectively). In our sensitivity analysis, the association between elevated depressive symptoms and non-adherence was of a similar magnitude and direction, although we were only able to assess unadjusted associations in this analysis due to small numbers of poor adherence and elevated depressive symptoms (OR 2.0; 95% CI 1.0–3.9, p = 0.06). Additional sensitivity analyses with cutoffs of CES-D ≥ 10 and CES-D ≥ 20 also resulted a statistically significant association between depressive symptoms and viral non-suppression (CES-D ≥ 10 aOR 2.1; 95% CI 1.3–3.3, p < 0.01; CES-D ≥ 20 aOR 2.4; 95% CI 1.2–4.5, p = 0.01). Among only those mothers who had a live birth, depressive symptoms were associated with greater odds of viral non-suppression, although this association was not statistically significant (aOR 1.7; 95% CI 1.0–3.1, p = 0.07). Based on this sample size, we had 89% power to detect an OR of 2.1 in our unadjusted GEE analysis.

**Table 2:** GEE analysis of depressive symptoms on viral non-suppression through the perinatal period

	Viral non- suppression	Unadjusted GEE analysis		Adjusted^a^ GEE analysis	
	N (%)^b^	OR (95% CI)	p-value	aOR (95% CI)	p-value
Depression status					
Elevated symptoms^c^	24 (29.6)	2.1 (1.3–3.7)	0.005	2.2 (1.2–4.0)	0.011
No elevated symptoms	194 (16.4)	Ref.	Ref.	Ref.	Ref.
Phase of perinatal period					
One-year pre-conception	120 (26.7)	4.5 (2.9–6.9)	< 0.001	4.7 (3.1–7.3)	< 0.001
Pregnancy	65 (17.4)	2.6 (1.7–4.0)	< 0.001	2.7 (1.8–4.3)	< 0.001
One-year postpartum	33 (7.5)	Ref.	Ref.	Ref.	Ref.

*GEE* generalized estimating equations; *OR* odds ratio; *aOR* adjusted odds ratio; *CI* confidence interval

^a^GEE models adjusted for study site and gravidity. Depressive symptom GEE analysis also adjusted for perinatal phase. Perinatal phase GEE analysis also adjusted for elevated depressive symptoms (CES-D ≥ 16)

^b^Number of visits with viral non-suppression out of total number of participant visits (n = 1263)

^c^Defined as CES-D ≥ 16

### Associations Between Depressive Symptoms, Viral Non-suppression and Birth Outcomes

**Fig. 2:**
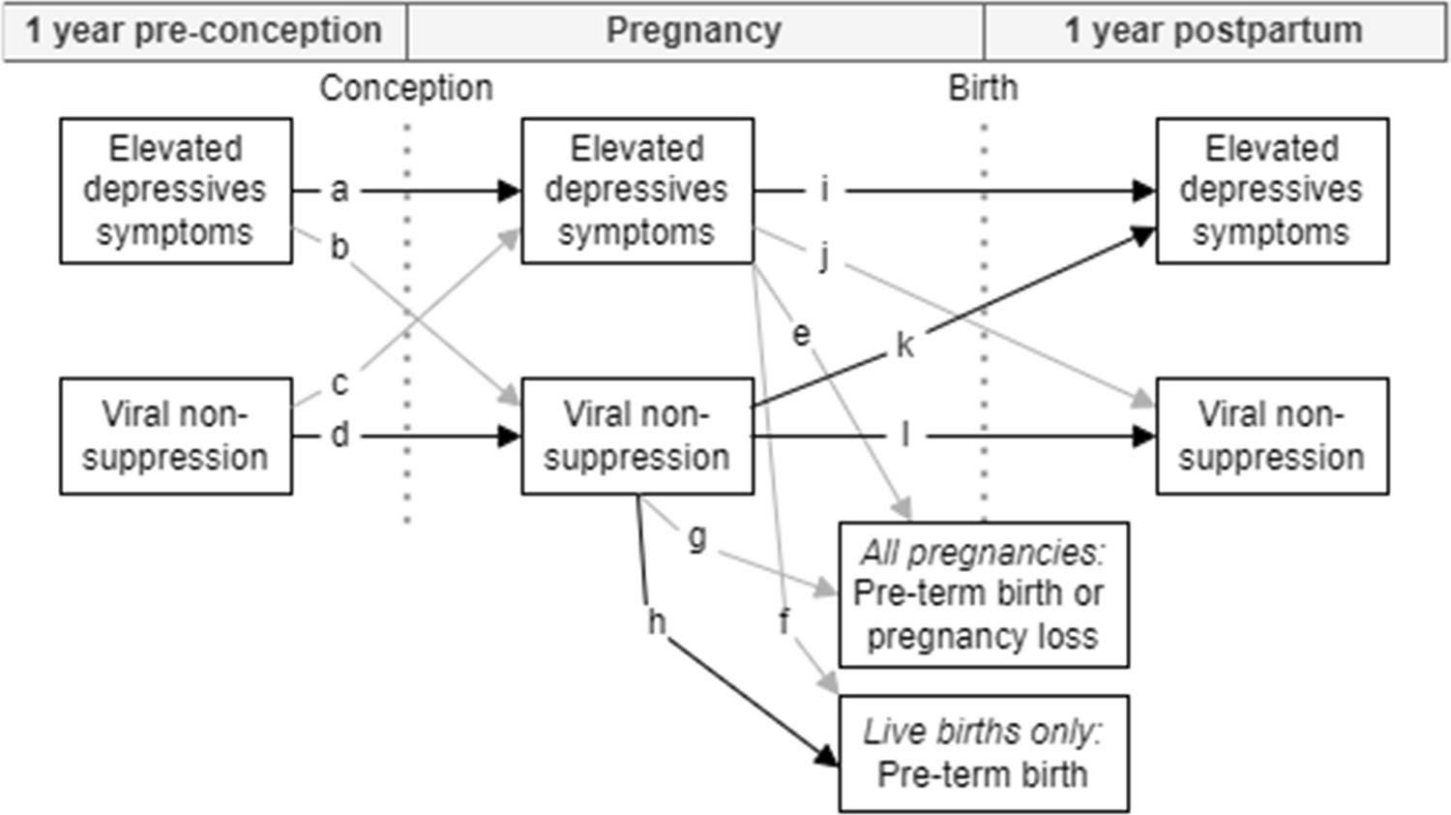
Associations between elevated depressive symptoms (CES-D ≥ 16), viral non-suppression (VL ≥ 200), and pre-term birth or pregnancy loss among WLWH in the perinatal period

Our second objective was to explore the relationships between depressive symptoms, viral non-suppression, and birth outcomes throughout the perinatal period in a series of unadjusted, participant-level, exploratory analyses (Fig. 2). Among women who had a live birth, viral non-suppression during pregnancy may be associated with greater odds of pre-term birth (OR 2.6; 95% CI 1.0–6.7; p = 0.05), although this association was not statistically significant at α = 0.05 (Table 3). Among all women who were pregnant, viral non-suppression during pregnancy was significantly associated with depression during the postpartum phase (OR 3.2; 95% CI 1.1–9.7, p = 0.04).

**Table 3:** Associations between elevated depressive symptoms (CES-D ≥ 16), viral non-suppression (VL ≥ 200), and pre-term birth or pregnancy loss among WLWH in the perinatal period

Model	Predictor	Outcome	OR (95% CI)	p
	1 year pre-conception	Pregnancy		
a	Elevated depressive symptoms	Elevated depressive symptoms	4.9 (1.8–13.0)	< 0.01
b	Elevated depressive symptoms	Viral non-suppression	1.8 (0.8–4.0)	0.13
c	Viral non-suppression	Elevated depressive symptoms	1.1 (0.4–2.8)	0.89
d	Viral non-suppression	Viral non-suppression	5.8 (3.0–11.2)	< 0.01

	Pregnancy	Pregnancy		
e	Elevated depressive symptoms	Pre-term birth or pregnancy loss	0.5 (0.2–1.8)	0.30
f	Elevated depressive symptoms	*Live births only*—pre-term birth	0.5 (0.1–4.0)	0.53
g	Viral non-suppression	Pre-term birth or pregnancy loss	1.3 (0.7–2.5)	0.47
h	Viral non-suppression	*Live births only*—pre-term birth	2.6 (1.0–6.7)	0.05

	Pregnancy	1 year postpartum		
i	Elevated depressive symptoms	Elevated depressive symptoms	5.4 (1.5–19.2)	0.01
j	Elevated depressive symptoms	Viral non-suppression	1.2 (0.3–5.5)	0.83
k	Viral non-suppression	Elevated depressive symptoms	3.2 (1.1–9.7)	0.04
l	Viral non-suppression	Viral non-suppression	10.6 (4.0–28.1)	< 0.01

## Discussion

Among WLWH who became pregnant during their participation in the AFRICOS cohort, approximately one fifth had elevated depressive symptoms (CES-D ≥ 16) at any point during their perinatal period, though few women, overall, had depressive symptoms at consecutive visits during their perinatal period. We found that, in the perinatal period, elevated depressive symptom levels were associated with doub1le the odds of viral non-suppression. This finding was robust to a number of sensitivity analyses including varying exposure definitions of depressive symptom severity, using non-adherence rather than viral non-suppression, and restricting to women who had a live birth. When we looked at timing of viral non-suppression and depression through the perinatal periods, we found that viral non-suppression in pregnancy was significantly associated with depressive symptoms postpartum. These findings have important implications for improving HIV and mental health care of women and timing of interventions.

The prevalence of depressive symptoms in this sample is lower than that reported in other studies of perinatal depression among HIV-positive women [20–23]. Studies using similar measures for depression among perinatal WLWH have found prevalence between 25% [62] and 50% [63, 64]. Meta-analyses of depression among perinatal WLWH found an average depression prevalence of 44% [21] and 36% [20]. Few WLWH in our sample had depressive symptoms throughout the perinatal period and, consistent with other studies, depressive symptoms were most prevalent during pre-conception and decreased by the postpartum period [20, 21]. There are several potential reasons for the lower prevalence of depressive symptoms observed in our study. First, the time since HIV diagnosis was longer among WLWH in this study—nearly 4 years—than among cohorts reported in the literature. This could have contributed to better HIV symptom control due to length of time since first ART prescription [65, 66] and lower risk of mental distress associated with recent diagnosis [67–69]. While some studies found higher rates of positive depression screening scores for women diagnosed with HIV during pregnancy [70]; the women included in the current study were engaged in HIV care prior to becoming pregnant. Second, the population in this analysis reported low rates of risk factors associated with perinatal depression—interpersonal violence (12%), poor literacy (12%) and never being married (20%)—which could have contributed to the low prevalence of depression [71]. Finally, pregnancy and children can be a motivator for treating depression [72], and adherence to ART improves among mothers who know their status before becoming pregnant [73]. The support structure of dedicated clinical HIV care could have positively influenced the participants’ risk of depression and detectable viral load. However, despite existing clinical support through participation in AFRICOS, depressive symptoms were associated with viral non-suppression.

Previous studies have found similar associations of depressive symptoms increasing risk for ART non-adherence or viral non-suppression, [28, 74, 75] but to our knowledge none has looked at this association across the perinatal period. For example, a previous analysis of the AFRICOS cohort showed that depressive symptoms reduced odds of ART adherence in adults (OR 0.59, p = 0.01) [40]. Another study of WLWH in Tanzania found that women with depression had 1.94 times the risk of viral non-suppression after 6 months of treatment, compared to women who had no depression [75]. We expected that our sensitivity analyses using a higher cut-off would result in a stronger magnitude of association between depressive symptoms and viral non-suppression [76]. Consistent with these hypotheses, we found twice the odds of viral non-suppression among mothers with the higher score indicating highly elevated depressive symptoms, strengthening our confidence in the association between depressive symptoms and viral non-suppression in this population. We also found that restricting the cohort to only mothers who had a live birth resulted in a weaker association (aOR 1.7; 95% CI 1.0–3.1, p = 0.07) between depressive symptoms and viral non-suppression, suggesting that poor outcomes of higher viral load, depression, and pregnancy loss may cluster together.

There are several biological and behavioral mechanisms that could explain our findings. Biologically, depression can alter the function of the body’s immune response and lead to an increase in viral load [28, 29]. Additionally, depression can increase risk of viral non-suppression through behavior changes that reduce ART adherence [26, 77–79], co-occurring alcohol use, and through poor social support [26, 79]. The emotional and behavioral effects of depression, including feelings of hopelessness, limited social interactions, and lack of motivation, can reduce adherence to ART [77, 79]. Our sensitivity analysis found increased, although not significant, odds of non-adherence among perinatal women with depressive symptoms (OR 2.0, p = 0.06). A previous study of pregnant women in PMTCT care in South Africa found that elevated depressive symptoms were directly associated with significantly lower adherence to ART [26]. Additional research is needed to assess the relative contribution of these mechanisms to the outcomes we observe.

We found that women who were virally non-suppressed had more than twice the odds of a pre-term birth in exploratory unadjusted analyses. Previous studies have found that pregnant women experiencing depression are up to 40% more likely to have a preterm birth, a relationship that is stronger in low- and middle-income countries [80–82]. Another study in Kenya found that risk of preterm birth was 3.6 times higher among pregnant women with depressive symptoms [83]. Moreover, WLWH are known to be more likely to have a preterm birth or baby with low birthweight than HIV-negative women [84–86]. One study in Botswana showed that pregnant women continuing highly active ART from before pregnancy had higher odds (aOR 1.4) of preterm delivery, compared to women who started ART in pregnancy after adjusting for maternal hypertension and anemia [87]. We also found significantly higher odds of elevated depressive symptoms postpartum among women who had viral non-suppression in their pregnancy. Previous studies have found that viral suppression is associated with a reduced odds of depression symptom severity among pregnant and postpartum women (aOR 0.45) [88]. One meta-analysis showed significantly increased odds of postpartum depressive symptoms among WLWH compared to women without HIV (OR 1.58) [20]. Biologically, indoleamine 2,3-dioxygenase-1 (IDO) is an enzyme that is induced both in HIV infection and post-partum depression and stimulates tryptophan catabolism, contributing to decreased T-cell proliferation and depletion of serotonin–factors that can lead to viral non-suppression and depressive symptoms [89]. Further research is needed to understand this relationship in more detail, including analyses with large sample sizes which would allow for mediation analyses using marginal structural models.

The 2018 Lancet Commission on strengthening the global HIV response highlighted the need to integrate HIV and other health care delivery [6]. The integration of mental health and HIV care is critical to ensure the wellbeing of WLWH and the health of children born to them. This study has several implications for policy and clinical intervention. Our robust findings of twice the odds of viral non-suppression among women with depressive symptoms during the perinatal period supports the inclusion of screening and care for mental disorders for pregnant women, women planning to become pregnant, and women of reproductive potential in general. Timing of screening and intervention is also an important consideration, as we found significant relationships between viral non-suppression and depressive symptoms from pregnancy through postpartum. Low adherence to ART and high viral load among pregnant WLHV who have depressive symptoms can increase risk of vertical transmission for pregnant mothers [77, 90]. The findings of this analysis support a growing body of research that advocates for the integration of mental health and HIV care programs to support WLWH of childbearing age. [91]. Several integrated mental health and adherence interventions (i.e. Life Steps) [92] have shown improvements in both depressive symptoms and adherence, but few have been implemented among pregnant populations [93]. The perinatal period, with multiple antenatal and child care visits, presents a unique opportunity to screen for and treat mental health conditions [94]. Integration of screening and care for mental health conditions can strengthen HIV prevention and care outcomes [1] in addition to perinatal mental health disorder treatments and ultimately support improved maternal and child outcomes [91].

A number of limitations of this analysis should be considered. First, pregnancy-related variables were all collected through chart review abstraction, and we were limited by the availability and quality of data. Data on antidepressant medication is likely incomplete and therefore could not be included as a covariate in our models. Antidepressant use, if data were available, would be a confounding factor in examining the association of prenatal depression with pregnancy outcome such as preterm birth. Accurate adjustment for this factor may decrease the strength of our association. In our analysis, there may be unmeasured confounders of the relationship between depression and viral non-suppression. Both depression and viral load are influenced by gender-based violence, empowerment, stigma, and other factors that were not measured in AFRICOS [95–98]. Additionally, pregnancy intention data was not recorded in this cohort study and unintended pregnancy is associated with transient elevated depression symptomatology [99]. We were unable to adjust for non-nucleoside reverse transcriptase inhibitors (NNRTIs) for HIV treatment (efavirenz, nevirapine) that have been associated with depressive symptoms and suicidal ideation [59], since most women (86%) were on an EFV/NVP regimen. Additionally, women in this study had clinic visits once every six months and were asked about their depressive symptoms for the previous two weeks. Therefore, we may not capture women who only experienced immediate postpartum depressive symptoms. However, there is growing evidence that perinatal depression is present throughout pregnancy and the year postpartum, beyond the first 6 weeks [45–47]. Finally, we are unable to draw conclusions about the causal relationship between depression and viral load from this analysis since we cannot rule out the potential bidirectional relationship, and we have identified several unmeasured and time-varying confounding factors. Additionally, in our secondary analysis we had few outcomes of elevated depressive symptoms and viral non-suppression at most time points, limiting our ability to explore causal pathways. Future work is needed to explore the temporality of these associations. Despite these limitations, our findings on the association between depressive symptoms and viral non-suppression were consistent across multiple sensitivity analyses modifying the analysis exposure, outcome, and population definitions. There are also several strengths to this analysis. We found high statistical power to detect the effect in our primary analysis. We included WLWH from a variety of countries and sites within countries, increasing potential generalizability of findings. The study followed women throughout each stage of the perinatal period with regular depression measurements and validated depression scales for HIV-positive pregnant women. High retention in the cohort through the perinatal stages reduced the risk of bias. Also, while most previous studies have looked at the association between depressive symptoms and HIV treatment outcomes using ART adherence measures as an outcome [78], our analysis utilized regularly collected viral load data as a clinical outcome. Finally, we also included a clear definition of the perinatal period and included the preconception period, which is rarely included in studies of the perinatal period.

## Conclusion

Our analysis showed an increased odds of viral non-suppression associated with depressive symptoms among perinatal women living with HIV. Future research is needed to further explore this relationship with a larger sample size and including a wider array of maternal and child clinical outcomes. These findings accentuate the need for screening and treatment of pregnant women with depressive symptoms in HIV care settings which has the potential to improve HIV, perinatal mental health, as well as birth outcomes.

## Data Availability

The Henry M. Jackson Foundation for the Advancement of Military Medicine (HJF) and the Water Reed Army Institute of Research (WRAIR) are committed to safeguarding the privacy of research participants. Distribution of data will require compliance with all applicable regulatory and ethical processes, including establishment and approval of an appropriate data-sharing agreement. To request a minimal data set, please contact the data coordinating and analysis center (DCAC) at PubRequest@hivresearch.org and indicate the RV329 study along with the name of the manuscript.

## Code Availability (Software Application or Custom Code)

Data analysis code can be made available upon request.
